# The effect of youths as change agents on self-rated health status and happiness among adult neighbors: a cluster randomized controlled trial in Sri Lanka

**DOI:** 10.3389/fpubh.2025.1649948

**Published:** 2025-09-01

**Authors:** Zobida Islam, Tetsuya Mizoue, Nadeeka Chandraratne, Susantha Indrawansa, Nalika Gunawardena

**Affiliations:** ^1^Department of Epidemiology and Prevention, Japan Institute for Health Security, Tokyo, Japan; ^2^Department of Community Medicine, Faculty of Medicine, University of Colombo, Colombo, Sri Lanka; ^3^Diabetes Research Unit, Faculty of Medicine, University of Colombo, Colombo, Sri Lanka; ^4^World Health Organization, Country Office for Sri Lanka, Colombo, Sri Lanka

**Keywords:** self-rated health, happiness, youth-led intervention, randomized controlled trial, Sri Lanka

## Abstract

**Background:**

Lifestyle modification intervention may influence the mental health of the participants. The present study examined the effect of youth-led interventions on self-rated health (SRH) and happiness among community adults in Sri Lanka.

**Methods:**

This cluster-randomized controlled trial study was conducted in a semi-urban area of Colombo in Sri Lanka. Out of 68 Grama Niladari (GN) divisions in the selected Divisional Secretariat, 24 GN (intervention = 12 and control = 12) were randomly selected. The intervention group received health education from their community’s young adults (aged 15–29 years) to identify and modify the determinants of their undesired health behaviors twice a month. The control group received no intervention. At baseline and at 12-month follow-up surveys, participants were asked to rate their SRH and happiness using a single-question measure. Multilevel logistic and multilevel linear regression models were used to assess the effects of the intervention on changes in binary and continuous SRH and happiness, respectively, from baseline to end line.

**Results:**

At the 12-month follow-up, 483 adults aged 27 to 65 years (intervention = 245; control = 238) completed the final assessments out of the 512 participants enrolled at baseline. Participants in the intervention group showed higher odds of achieving good SRH [odds ratio (OR) 1.85, 95% confidence interval (CI) 1.18–2.90] compared to those in the control group. They showed a significantly greater change in SRH than controls; the mean difference in SRH change between the two groups was 0.13 (95% CI, 0.002–0.26). There was no significant difference in happiness between the two groups; the OR (95% CI) was 1.37 (0.85–2.22) for the intervention group.

**Conclusion:**

The results suggest that a youth-led educational program promoting healthier behaviors among their neighbors can effectively improve the SRH of community adults in a semi-urban area of Colombo, Sri Lanka.

**Clinical trial registration:**

https://slctr.lk/trials/slctr-2017-002, SLCTR/2017/002.

## Introduction

Noncommunicable diseases (NCDs) such as cardiovascular diseases (CVD), cancer, chronic respiratory diseases, and diabetes are the leading causes of death globally, posing a significant threat to public health. Each year, approximately 41 million people die due to NCDs, accounting for 7 out of every 10 deaths worldwide (1). Alongside NCDs, mental health disorders like depression and anxiety are also on the rise and present a substantial global health challenge. In 2019, the World Health Organization reported that an estimated 970 million people experienced a mental health issue, with 82% of these individuals living in low- and middle-income countries (2).

As a low- and middle-income country, although Sri Lanka has shown impressive progress in health indicators over the past decades, it continues to face a substantial burden of NCDs or lifestyle-related diseases (75% of the total deaths 40% were directly attributed to CVD) (3). Along with physical health, Sri Lanka is lagging way behind in terms of mental health. Despite the fall in rates of suicide since the mid-1990s, the rate of suicide still remained high (14.0% per 100,000 population) (4). Furthermore, mental health has been deprioritized in Sri Lanka’s healthcare system for a long period of time and is rarely a topic that is prioritized or talked about. Preventive initiatives raising public awareness to identify and modify the risk factors of both physical health and mental health are necessary to improve overall health status in this resource-scarce area.

Professional healthcare workers have been shown to play a critical role in promoting healthy lifestyles and preventing NCDs (5, 6). However, low—and middle-income countries are projected to face a shortfall of 10 million healthcare workers by 2030, necessitating alternative approaches to healthcare delivery (7, 8). Non-professional healthcare workers, such as community health workers, have shown promise in addressing these challenges (9, 10).

Additionally, school-aged children and youth have been demonstrated to act as change agents in promoting healthy behaviors among their parents. Intervention studies in Brazil (11) and northern China (12) have shown that providing health education to school-aged children and training them to act as change agents effectively promotes healthy behaviors among their parents. In Sri Lanka, engaging school children as change agents for promoting healthy habits among their mothers has proven to be effective in reducing body weight and increasing physical activity (13). In our previous intervention study, where youth acted as a change agent, a significant reduction in body weight was observed in the intervention group compared to the control group (14). While the primary goal of such interventions is to reduce NCD risk factors through healthier behaviors, there is increasing evidence that lifestyle improvement can also enhance mental well-being, including happiness (15–17). Happiness is an important component of subjective well-being, linked to better health behaviors and improved self-rated health (SRH) (18). Therefore, assessing happiness as an outcome alongside SRH provides a more holistic understanding of the broader benefits of an intervention program focusing on lifestyle changes.

Focusing on a 12-month community-based intervention program in Sri Lanka that empowers young individuals to act as change agents in promoting healthy lifestyles among community adults to reduce NCD risks, this study aimed to assess the impact of these youth-led initiatives on SRH status and happiness among community adults.

## Materials and methods

### Study design

**Figure 1:**
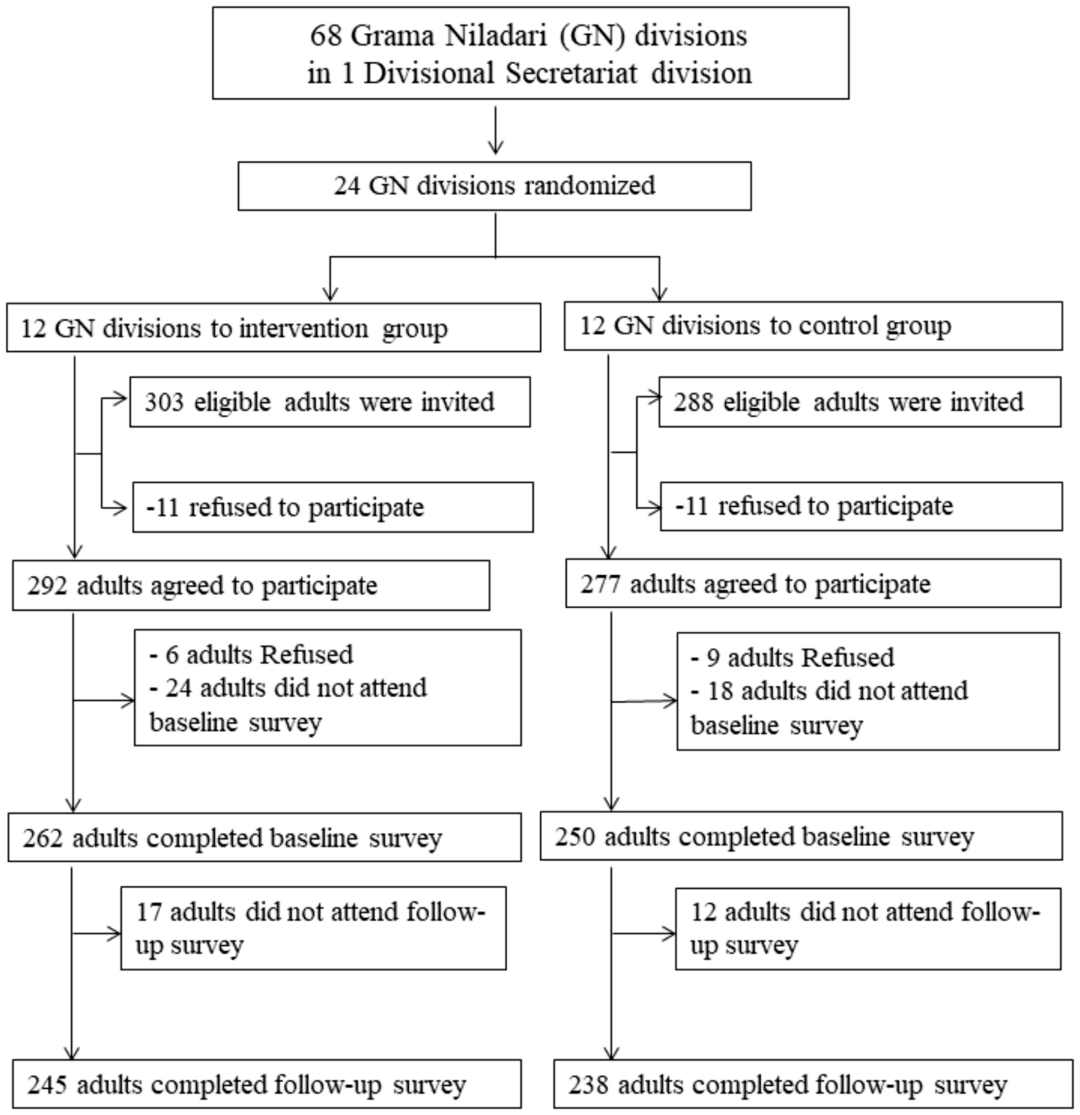
Study flow diagram.

This study is a secondary analysis of a 12-month cluster randomized controlled trial conducted in a semi-urban area of Colombo, Sri Lanka. This intervention trained youths, who were members of youth clubs, to act as change agents in promoting healthy lifestyles among their neighbors, aiming to reduce CVD risk factors. The intervention study was conducted in one eligible Divisional Secretariat division in the district of Colombo, which is subdivided into 68 Grama Niladari (GN) divisions with one youth club on average per GN division. Of the 68 GN divisions, 24 (12 for intervention and 12 for control) were selected using a random procedure. In the baseline survey (April-August 2016), 591 eligible participants (303 in the intervention group and 288 in the control group) were invited. Of them, 512 participants (262 in the intervention group and 250 in the control group) completed the baseline survey (Figure 1). During the intervention, 45 young adults (aged 15–29 years), who were trained by the facilitators of the Foundation of Health Promotion (Sri Lanka) and acted as change agents, visited the intervention area twice a month to propose healthier lifestyle choices to adults and encourage them to adopt healthier behaviors. The youth staff monitored the progress of health promotion for their respective neighbors and reported at least once per month to the facilitators. On the other hand, the control group received no intervention. One year after the initiation of the intervention, an endline assessment was conducted. Detailed information about the selection procedure of the GN division, the target population, intervention, sample size estimation, and the results of the primary outcomes was described in our previous publication (14). The study protocol was approved by the Ethics Review Committee of the Sri Lanka Medical Association and the Ethics Committee of the National Center for Global Health and Medicine, Japan. All participants provided written consent before the study. The study was registered in the Sri Lanka Clinical Trials Registry (SLCTR/2017/002).

#### Outcomes

The outcome measures in this study were SRH and happiness. SRH was assessed using a single question: “In general, would you say your health is…” and response options were “excellent to poor.” Following the previous studies (17, 18), the response options “excellent,” “very good,” and “good” were categorized as “good,” and the options “fair” and “poor” were categorized “poor” in this study.

Happiness was assessed using a single question: “Considering the past month which describes your psychological status best?” and response options were “(1) very happy, (2) happy, (3) moderate, (4) unhappy/sad/stressed, and (5) very sad/stressed.” In this study, the responses “very happy” and “happy” were classified as “happiness,” whereas “moderate,” “unhappy/sad/stressed,” and “very sad/stressed” were classified as “unhappiness.”

#### Measurements

Health surveys were conducted at baseline and after the intervention according to the WHO STEPS protocol (19). Trained youth, who were not involved in the intervention and were unaware of the community allocations, collected data at both baseline and follow-up after receiving a half-day training that included a lecture and practical exercises on interview techniques and physical measurements. Following a survey questionnaire, the young staff collected information on sociodemographic characteristics (age, sex, ethnicity, religion, education, and household income) and health-related behaviors, including smoking status, frequency and quantity of alcohol consumption, daily intake of fruits and vegetables (frequency and servings), weekly consumption of snacks and sugar-sweetened beverages, and duration of work-related and leisure-time physical activity. They measured the height and weight to the nearest 0.1 cm and 0.2 kg, using a portable stadiometer (Seca 213; Seca Ind., Hamburg, Germany) and a digital weighing scale (WB220; Rossmax, Berneck, Switzerland), while participants were wearing light clothing and no shoes. To calculate body mass index (BMI), body weight (in kg) was divided by the square of the height (in meters).

### Statistical analysis

The analysis included the participants who completed both baseline and follow-up surveys. Socio-demographic and outcome variables were presented as mean ± standard deviation (SD) for continuous data and as number (percentage) of participants for categorical data. To investigate the effect of the intervention on SRH and happiness, a multilevel logistic regression model was employed, comparing the good and happiness status to the poor and unhappiness status. In addition, a multilevel linear regression model was used to assess the mean changes in SRH and happiness. For this approach, SRH and happiness were treated as continuous variables because even when assessed with five response options, they form a continuum from poor to good and allow for an increase in the explained variance (20–22). Two-level of multilevel models were used to account for the clustering of neighbors within each GN division as a random effect in addition to the individual level as a fixed effect (23). The models were adjusted for the baseline value of both categorical and continuous outcomes in order to take into account the difference at baseline. The multilevel models were considered as follows:

#### Multilevel logistic regression

$$\begin{array}{l} \text{logit}\;\mathrm{Pr}\;({\text{binary outcomes}}_{\mathrm{ij},\text{follow}\;\mathrm{up}})=\mu +b_{j}\\ +\alpha \cdot {\text{intervention}}_{\mathrm{ij}}+\beta \cdot {\text{binary outcomes}}_{\mathrm{ij},\text{baseline}} \end{array}$$

#### Multilevel linear regression

$$\begin{array}{l} {\text{Outcomes}}_{\mathrm{ij},\text{followup}}-{\text{Outcomes}}_{\mathrm{ij},\text{baseline}}=\mu +b_{j}\\ +\alpha \cdot {\text{intervention}}_{\mathrm{ij}}+\beta \cdot {\text{continuous outcomes}}_{\mathrm{ij},\text{baseline}} \end{array}$$

where the probability of individual *i* in GN division *j* having a given outcome at the end of the follow-up (follow-up) was modeled by intervention assignment (intervention group or control group) and outcome at baseline, (baseline) was the control of outcomes at baseline, μ was the intercept, and (*bj*) was the random intercepts of GN divisions. The adjusted odds ratio (OR) from the logistic regression model and the mean from the linear regression model were calculated for the binary and continuous outcomes, respectively, along with their 95% confidence intervals (CIs). We also performed a series of sensitivity and subgroup analyses to assess the robustness of the intervention effects. Previous studies have shown that overweight/obese adults are more likely to report poor SRH (24) and that a reduction of one BMI unit among obese individuals is associated with an increased likelihood of reporting good SRH (25). We therefore repeated the analyses after restricting the intervention group to participants who were overweight/obese (BMI ≥ 25 kg/m^2^) at baseline and lost ≥2 kg by endline (*n* = 63), while retaining the full control group for comparison. Similarly, we assessed whether the intervention effects differed when the intervention group was restricted to those who reported increased vegetable or fruit intake from baseline to endline (*n* = 139). We also investigated the intervention effect on SRH among those who had poor SRH at baseline (intervention group = 157; control group = 136), and on happiness among those who were unhappy at baseline (intervention group = 151; control group = 129). Statistical significance was set at *p* < 0.05 for trend. All analyses were performed using the statistical software Stata version 18.0 (StataCorp, College Station, Texas, United States).

## Result

As shown in Figure 1, of the 24 GN divisions, 591 eligible households (*n* = 303 for the intervention group and *n* = 288 for the control group) were invited to the baseline survey, of which 22 households (*n* = 11 from each group) refused to participate, leaving 569 adults (*n* = 292 for intervention and *n* = 277 for control) who agreed to participate in the baseline survey. Later, 15 adults refused to participate (*n* = 6 for intervention and *n* = 9 for control), and 42 adults (*n* = 24 for intervention and *n* = 18 for control) did not attend the baseline survey, leaving 512 adults (*n* = 262 from the intervention group and *n* = 250 from the control group) who completed the baseline survey. At the 12-month follow-up, 29 adults (*n* = 17 from the intervention group and *n* = 12 from the control group) did not attend the survey, leaving 483 adults (*n* = 245 from the intervention group and *n* = 238 from the control group) for the complete analyses.

Table 1 shows the baseline characteristics of the study participants. The mean (±SD) age was 46.1 ± 8.1 years in the intervention group and 44.8 ± 8.2 years in the control group. The intervention and control groups were similar in terms of sex, ethnicity, religion, socioeconomic status, BMI, fruit consumption, snack consumption, sugar-sweetened beverage consumption, and smoking. However, the intervention group had lower proportions of current workers, vegetable intake, and a history of dyslipidemia but higher proportions of participants who were physically active, had a history of hypertension or diabetes, and reported good SRH and happiness than the control group.

**Table 1:** Baseline characteristics of study participants.

Characteristics	Control group	Intervention group
Number of participants	238	245
Age (mean ± SD, Y)	44.8 (±8.2)	46.1(±8.1)
Age categories, years (*n*, %)		
<35	32 (13.0)	27 (11.0)
35 to <45	87 (36.6)	75 (30.6)
45 to <55	83 (34.9)	98 (40.0)
≥55	36 (15.1)	45 (18.3)
Sex (women, %)	124 (52.1)	130 (53.1)
Ethnicity (Sinhalese, %)	226 (95.0)	234 (95.5)
Religion (Buddhism, %)	218 (91.6)	218 (89.0)
Educational attainment (high school or higher, %)	59 (24.8)	66 (26.9)
Household income (≥60,001 Rupees/month, %)	13 (5.5)	13 (5.3)
Current workers (yes, %)	137 (57.6)	123 (50.2)
BMI (mean ± SD, kg/m^2^)	24.9 (± 4.6)	25.8 (± 4.9)
BMI categories (*n*, %)		
Underweight (<18.5 kg/m^2^)	20 (8.4)	21 (8.6)
Normal weight (18.5–24.9 kg/m^2^)	103 (43.3)	110 (44.9)
Overweight (25–29.9 kg/m^2^)	86 (36.1)	85 (34.7)
Obese (≥30 kg/m^2^)	29 (12.2)	29 (11.8)
Physical activity (≥150 min/week of moderate-intensity or ≥75 min/week of vigorous-intensity physical activity, %)	192 (80.7)	210 (85.7)
Alcohol intake (low risk of drinking level, %)*	221 (92.9)	237 (96.7)
Fruits consumption (≥2 servings/day, %)	19 (8.0)	20 (8.2)
Vegetable consumption (≥3 servings/day, %)	90 (37.8)	81 (33.1)
Snacks consumption (<twice/day, %)	147 (61.8)	155 (63.3)
Sugar-sweetened beverage consumption (<once/day, %)	223 (93.7)	225 (91.8)
Smoking status (never or former, %)	202 (84.9)	207 (84.5)
History of diabetes (yes, %)	39 (16.4)	42 (17.1)
History of dyslipidemia (yes, %)	33 (13.9)	25 (10.2)
History of hypertension (yes, %)	47 (19.8)	55 (22.5)
SBP (mean ± SD, mmHg)	127.3 (±18.8)	126.7 (±20.7)
DBP (mean ± SD, mmHg)	84.4 (±10.9)	83.6 (±12.6)
Outcomes		
Self-rated health (mean ± SD)	2.38 (±0.82)	2.28 (±0.81)
Self-rated health categories (*n*, %)		
Excellent	6 (2.5)	4 (1.6)
Very good	5 (2.1)	8 (3.3)
Good	91 (38.2)	76 (31.0)
Fair	108 (45.4)	122 (49.8)
Poor	28 (11.8)	35 (14.3)
Happiness in the past month (mean ± SD)	3.42 (±0.70)	3.34 (±0.70)
Happiness in the past month categories (*n*, %)		
Very happy	10 (4.2)	11 (4.5)
Happy	99 (41.6)	83 (33.8)
Moderate	110 (46.2)	129 (52.7)
Unhappy	19 (8.0)	22 (9.0)
Very sad	0	0

BMI, body mass index; SD, standard deviation; SBP, systolic blood pressure; DBP, diastolic blood pressure.

Data are presented as numbers (percentages) unless otherwise indicated.

*Low risk of drinking level was defined as two drinks/day for men and one drink/day for women.

As shown in Table 2, at baseline, 35.9% of participants in the intervention group and 42.9% in the control group reported good SRH. At follow-up, these figures changed to 55.9% in the intervention group and 45.8% in the control group. The participants in the intervention group showed significantly higher odds of achieving good SRH at the 12-month follow-up compared to the control group; the OR (95% CI) was 1.85 (1.18–2.90) (*p* = 0.01) for the intervention group. Regarding happiness, 38.4% of participants in the intervention group and 45.8% in the control group reported happiness at baseline. These figures changed to 45.7% in the intervention group and 39.9% in the control group at follow-up. However, the odds of happiness were not significant for the intervention group compared to the control group at the 12-month follow-up; the OR (95% CI) was 1.37 (0.85–2.22) (*p* = 0.20) for the intervention group after adjusting for the baseline happiness status.

**Table 2:** Effect of intervention on self-rated health and happiness at 12-month follow-up.

Outcome	Intervention group (*n* = 245)		Control group (*n* = 238)		Between intervention and control group at follow-up^$^	*p*-value
	Number (%) at baseline	Number (%) at follow-up	Number (%) at baseline	Number (%) at follow-up	OR (95% CI)	
Self-rated health						
Good	88 (35.9)	137 (55.9)	102 (42.9)	109 (45.8)	**1.85 (1.18–2.90)**	**0.01**
Happiness in the past months						
Happy	94 (38.4)	112 (45.7)	109 (45.8)	95 (39.9)	1.37 (0.85–2.22)	0.20

OR, odds ratios; CI, confidence interval.

^$^Multilevel logistic regression, with Grama Niladari divisions as the cluster variable and adjustment for each outcome variable at baseline. The bold values indicate statistical significance.

Table 3 shows the mean changes in SRH and happiness from baseline to 12-month follow-up when SRH and happiness are treated as continuous variables. In the multilevel linear regression analysis considering GN divisions as the cluster, the intervention group showed significantly positive changes compared to the control group; the mean (95% CI) of changes in the difference between the two groups was 0.13 (0.002–0.26) (*p* = 0.046). As for the results of happiness in the past month, the changes in the difference between the two groups were not statistically significant; the mean (95% CI) of changes in the difference between the two groups was 0.09 (−0.04–0.21) (*p* = 0.18).

**Table 3:** Changes in self-rated health and happiness from baseline to 12-month follow-up.

Outcomes	Intervention group (*n* = 245)		Control group (*n* = 238)		Between intervention and control group at follow-up^c^	
	Mean ± SD at the end of the follow-up	Mean ± SD change from the baseline^a^	Mean ± SD at the end of the follow-up	Mean ± SD change from baseline^a^	Difference in mean (95% CI)^b^	*p* value
Self-rated health						
Good	2.54 ± 0.74	0.26 ± 0.89	2.45 ± 0.76	0.07 ± 0.75	**0.13 (0.002–0.26)**	**0.046**
Happiness in the past months						
Happy	3.44 ± 0.63	0.10 ± 0.86	3.37 ± 0.60	−0.05 ± 0.83	0.09 (−0.04 to 0.21)	0.18

CI, confidence interval; SD, standard deviation.

a Change from baseline = outcome values at the end of the follow-up—outcome values at baseline.

b The calculated outcome of “change from the baseline” was used for the analysis of “between group differences at follow-up.”

c Multilevel linear regression, with Grama Niladari divisions as the cluster variable and adjustment for each outcome variable at baseline. The bold values indicate statistical significance.

Regarding the results of sensitivity analyses, the positive effect of the intervention on SRH remains materially unchanged among those in the intervention groups who were overweight/obese at baseline and lost ≥2 kg weight at end-line (Supplementary Table S1), those who increased their fruit or vegetable consumption at the follow-up (Supplementary Table S2), and among those who reported poor SRH at baseline (Supplementary Table S3). For happiness, no significant association was observed in the analyses restricted to participants who were overweight/obese and lost ≥2 kg (Supplementary Table S1) or to those who were unhappy at baseline (Supplementary Table S4). However, a significant positive association with happiness was found among participants who increased their fruit or vegetable consumption from baseline to follow-up (Supplementary Table S2).

## Discussion

This randomized controlled trial study found that the intervention group showed a statistically significant improvement in SRH than the control group at 12-month follow-up. However, while the intervention group showed some improvement in happiness at the end of follow-up, there was no significant difference in happiness between the intervention and control groups.

The present findings on the beneficial effect of lifestyle intervention on SRH are supported by two previous interventions conducted among middle-aged adults in the UK (26) and Finland (27), but not by those conducted among young adolescent men in Finland (28) and older adults in Israel (29). The precise reason for the inconsistent results among studies is not clear, but it can be attributed to the differences in participants’ background characteristics, intervention strategies, and the duration of the interventions. For instance, the interventions in Finland (28) and Israel (29) lasted 6 and 3 months, respectively—substantially shorter than the 12-month durations in our study and the studies conducted in the UK (26) and Finland (27). A shorter intervention period might not provide sufficient time for participants to experience or report noticeable changes in their health. Additionally, compared to the previous studies (26–30), the strategy of the current intervention study was unique, as it provided health education to the young people who were responsible for promoting and modifying their neighbors’ lifestyles. The beneficial effect of the intervention on SRH in our study might be attributed to the improvement of the lifestyle within the intervention group, such as reduced body weight (−2.83 kg), increased fruit consumption (OR 1.71), and reduced snack consumption (OR 0.32), and increased, although not statistically significant, leisure-time physical activities (OR 1.58), compared to the control group (14). These changes may contribute to the improvement of the SRH (31, 32). Furthermore, participation in outdoor activities or games with other adults or their children not only enhanced physical health but also provided a chance to communicate with their neighborhood and community people and to exchange their emotions. Additionally, contact and communication with youth club members through the intervention might have contributed to the improvement of mental well-being. Together with the above, the current study suggests that the approach targeting youth to promote healthy lifestyles may improve adults’ SRH in Sri Lanka.

Data on the effect of lifestyle intervention on the level of happiness is scarce. An intervention study targeting weight loss maintenance and psychological well-being used a mobile phone app (MotiMate) to promote lifestyle changes among Australian adults through features such as tracking weight, food intake, and physical activity, alongside mood and stress workshops (33). Participants in the intervention group also received additional support from dietitians or psychologists when needed, particularly for weight gain or highly negative moods. At the 24-week follow-up, the study found an improvement in happiness, albeit statistically not significant, between the intervention and control groups (who used an app with monitoring features only, excluding mood and stress) (33). Another intervention study with green space use and happiness as primary outcomes among Hong Kong high school students integrated hydroponic planting with health promotion activities, such as lessons on balanced lifestyles, healthy eating, and physical exercise, over 6 weeks (34). This study showed a significant improvement in happiness among the intervention group compared to the control group after participating only in health promotion activities (34). Unlike the Australian (33) and Hong Kong (34) studies, which incorporated psychological well-being components into their intervention, our study was primarily focused on reducing CVD risk by enabling young people through health education programs to promote pure healthy behavior changes in their communities. Therefore, extra attention was not given to the psychological issues, such as stress management, mindfulness activities, or emotional support, in the intervention group. This limited focus may partly explain the non-significant effect on happiness, suggesting that lifestyle behavior change alone may not be sufficient to improve psychological well-being. Further research with more extensive material, including psychological intervention components (i.e., exchanging emotions, gratitude, life satisfaction, mindfulness) is needed.

The strengths of the present study include its cluster-randomized controlled design, which helps to prevent the influence of measured and unmeasured confounders, increasing the internal validity of the study. The study presents an innovative approach to community health promotion, harnessing the potential of young adults as agents of change. This demonstrates a scalable and sustainable strategy for improving population health in resource-limited settings. The present study also has several limitations that warrant mention. First, information on SRH and happiness in the past month was assessed through interviews, which raises concerns about interviewer bias and subjective reporting. Although the interviewers were blinded to intervention allocation, the subjective nature of these self-reported measures and the direct interaction may introduce some bias. Second, the sample size of the study was calculated for the primary outcome of the original study (body weight) and may not have been sufficiently powered to detect a modest effect on other outcomes, such as happiness. Third, SRH and happiness were assessed only at baseline and 12-month follow-up. More frequent assessments could have captured the dynamic changes in health and well-being over time. Fourth, the happiness scale used in the survey was created specifically for the present intervention study, without confirming quality assurance, such as ensuring face validity (35, 36). Using this single-item, non-validated measure may limit the reliability and interpretability of our findings related to happiness, as it does not capture the multidimensional nature of this construct. To strengthen the assessment of happiness, future studies should consider using validated scales, such as the Memorial University of Newfoundland Scale of Happiness, the Oxford Happiness Questionnaire, or the Subjective Happiness Scale. Fifth, this study was unable to clarify the long-term impact of the intervention program beyond 12 months. Sixth, although the intention-to-treat method was applied (37), the exclusion of refusals and non-attendance at the follow-up survey from all randomized subjects may introduce some bias. Seventh, study participants and assessors (youth club members) were not masked about their intervention status, which could lead to response bias in the intervention group (exaggerating the favorable aspects). Finally, the intervention study was performed in one Divisional Secretariat out of 13 Divisional Secretariats of the semi-urban area of Colombo. Therefore, the applicability of this program to other urban and rural areas of Sri Lanka remains unclear, and caution should be exercised when generalizing these results to groups with different backgrounds.

## Conclusion

An educational program that utilized youth to encourage healthier behaviors among neighbors improved the SRH of community adults in a semi-urban area of Colombo, Sri Lanka. This study provides evidence that lifestyle interventions for preventing NCDs contribute to the mental well-being of people in low- and middle-income countries. Further studies are needed to develop a comprehensive intervention that incorporates a psychological component and confirms its impact on physical and mental well-being.

## Data Availability

The raw data supporting the conclusions of this article will be made available by the authors, without undue reservation.

## References

[1] World Health Organization. Noncommunicable diseases. Available online at: https://www.who.int/news-room/fact-sheets/detail/noncommunicable-diseases (Accessessed November 21).

[2] World Health Organization. World mental health report: Transforming mental health for all. Geneva: World Health Organization (2022) Licence: CC BY-NC-SA 3.0 IGO.

[3] Forouzanfar MH Afshin A Alexander LT Anderson HR Bhutta ZA Biryukov S. Global, regional, and national comparative risk assessment of 79 behavioural, environmental and occupational, and metabolic risks or clusters of risks, 1990–2015: a systematic analysis for the global burden of disease study 2015. Lancet. (2016) 388:1659–724. doi: 10.1016/S0140-6736(16)31679-8, PMID: 27733284 PMC5388856

[4] World Health Organization Mental health atlas 2020 country profile: Sri Lanka. Available online at: https://www.who.int/publications/m/item/mental-health-atlas-lka-2020-country-profile (2020).

[5] Jeet G Thakur JS Prinja S Singh M. Community health workers for non-communicable diseases prevention and control in developing countries: evidence and implications. PLoS One. (2017) 12:e0180640. doi: 10.1371/journal.pone.0180640, PMID: 28704405 PMC5509237

[6] Khetan AK Purushothaman R Chami T Hejjaji V Madan Mohan SK Josephson RA. The effectiveness of community health workers for CVD prevention in LMIC. Glob Heart. (2017) 12:233–243.e6. doi: 10.1016/j.gheart.2016.07.001, PMID: 27993594

[7] Babagoli MA Nieto-Martínez R González-Rivas JP Sivaramakrishnan K Mechanick JI. Roles for community health workers in diabetes prevention and management in low-and middle-income countries. Cad Saude Publica. (2021) 37:e00287120. doi: 10.1590/0102-311X00287120, PMID: 34730688

[8] Abdel-All M Putica B Praveen D Abimbola S Joshi R. Effectiveness of community health worker training programmes for cardiovascular disease management in low-income and middle-income countries: a systematic review. BMJ Open. (2017) 7:e015529. doi: 10.1136/bmjopen-2016-015529, PMID: 29101131 PMC5695434

[9] Saks M. The regulation of healthcare professions and support workers in international context. Hum Resour Health. (2021) 19:74. doi: 10.1186/s12960-021-00618-8, PMID: 34103060 PMC8185486

[10] Schneider H Okello D Lehmann U. The global pendulum swing towards community health workers in low-and middle-income countries: a scoping review of trends, geographical distribution and programmatic orientations, 2005 to 2014. Hum Resour Health. (2016) 14:1–12. doi: 10.1186/s12960-016-0163-2 27784298 PMC5081930

[11] Fornari LS Giuliano I Azevedo F Pastana A Vieira C Caramelli B. Children first study: how an educational program in cardiovascular prevention at school can improve parents’ cardiovascular risk. Eur J Prev Cardiol. (2013) 20:301–9. doi: 10.1177/2047487312437617, PMID: 22345689

[12] He FJ Wu Y Feng XX Ma J Ma Y Wang H. School based education programme to reduce salt intake in children and their families (school-EduSalt): cluster randomised controlled trial. BMJ. (2015) 350:h770. doi: 10.1136/bmj.h770, PMID: 25788018 PMC4364292

[13] Gunawardena N Kurotani K Indrawansa S Nonaka D Mizoue T Samarasinghe D. School-based intervention to enable school children to act as change agents on weight, physical activity and diet of their mothers: a cluster randomized controlled trial. Int J Behav Nutr Phys Act. (2016) 13:45. doi: 10.1186/s12966-016-0369-7, PMID: 27048282 PMC4822262

[14] Chandraratne N Yamaguchi M Indrawansa S Gunawardena N Kuwahara K Islam Z. The effect of youths as change agents on cardiovascular disease risk factors among adult neighbours: a cluster randomised controlled trial in Sri Lanka. BMC Public Health. (2019) 19:893. doi: 10.1186/s12889-019-7142-1, PMID: 31286931 PMC6613264

[15] Haapasalo V de Vries H Vandelanotte C Rosenkranz RR Duncan MJ. Cross-sectional associations between multiple lifestyle behaviours and excellent well-being in Australian adults. Prev Med. (2018) 116:119–25. doi: 10.1016/j.ypmed.2018.09.003, PMID: 30218725

[16] Velten J Lavallee KL Scholten S Meyer AH Zhang X-C Schneider S. Lifestyle choices and mental health: a representative population survey. BMC Psychol. (2014) 2:1–11. doi: 10.1186/s40359-014-0055-y, PMID: 25628891 PMC4304169

[17] Zajacova A Huzurbazar S Todd M. Gender and the structure of self-rated health across the adult life span. Soc Sci Med. (2017) 187:58–66. doi: 10.1016/j.socscimed.2017.06.019, PMID: 28654822 PMC5554534

[18] Zarini GG Vaccaro JA Canossa Terris MA Exebio JC Tokayer L Antwi J. Lifestyle behaviors and self-rated health: the living for health program. J Environ Public Health. (2014) 2014:315042. doi: 10.1155/2014/315042, PMID: 25530764 PMC4228703

[19] World Health Organization. Stepwise approach to NCD risk factor surveillance (STEPS). Geneva: WHO (2021).

[20] Manderbacka K Lahelma E Martikainen P. Examining the continuity of self-rated health. Int J Epidemiol. (1998) 27:208–13. doi: 10.1093/ije/27.2.208, PMID: 9602400

[21] Perneger TV Gayet-Ageron A Courvoisier DS Agoritsas T Cullati S. Self-rated health: analysis of distances and transitions between response options. Qual Life Res. (2013) 22:2761–8. doi: 10.1007/s11136-013-0418-5, PMID: 23615958

[22] Cullati S Bochatay N Rossier C Guessous I Burton-Jeangros C Courvoisier DS. Does the single-item self-rated health measure the same thing across different wordings? Construct validity study. Qual Life Res. (2020) 29:2593–604. doi: 10.1007/s11136-020-02533-2, PMID: 32436111 PMC7434800

[23] Twisk J Bosman L Hoekstra T Rijnhart J Welten M Heymans M. Different ways to estimate treatment effects in randomised controlled trials. Contemp Clin Trials Commun. (2018) 10:80–5. doi: 10.1016/j.conctc.2018.03.008 29696162 PMC5898524

[24] Sung E-S Choi CK Jeong J-A Shin M-H. The relationship between body mass index and poor self-rated health in the south Korean population. PLoS One. (2020) 15:e0219647. doi: 10.1371/journal.pone.0219647, PMID: 32822339 PMC7442249

[25] Hafner L Tauchmann H Wübker A. Does moderate weight loss affect subjective health perception in obese individuals? Evidence from field experimental data. Empir Econ. (2021) 61:2293–333. doi: 10.1007/s00181-020-01971-8

[26] Steptoe A Perkins-Porras L Hilton S Rink E Cappuccio FP. Quality of life and self-rated health in relation to changes in fruit and vegetable intake and in plasma vitamins C and E in a randomised trial of behavioural and nutritional education counselling. Br J Nutr. (2004) 92:177–84. doi: 10.1079/BJN20041177, PMID: 15231001

[27] Engberg E Liira H Kukkonen-Harjula K From S Kautiainen H Pitkälä K. The effects of health counseling and exercise training on self-rated health and well-being in middle-aged men: a randomized trial. J Sports Med Phys Fitness. (2016) 57:916–22. doi: 10.23736/S0022-4707.16.06278-2 27045739

[28] Pyky R Koivumaa-Honkanen H Leinonen A-M Ahola R Hirvonen N Enwald H. Effect of tailored, gamified, mobile physical activity intervention on life satisfaction and self-rated health in young adolescent men: a population-based, randomized controlled trial (MOPO study). Comput Hum Behav. (2017) 72:13–22. doi: 10.1016/j.chb.2017.02.032

[29] Gabizon H Press Y Volkov I Melzer I. The effects of Pilates training on balance control and self-reported health status in community-dwelling older adults: a randomized controlled trial. J Aging Phys Act. (2016) 24:376–83. doi: 10.1123/japa.2014-0298, PMID: 26540737

[30] Rana AKMM Kabir ZN Lundborg CS Wahlin A. Health education improves both arthritis-related illness and self-rated health: an intervention study among older people in rural Bangladesh. Public Health. (2010) 124:705–12. doi: 10.1016/j.puhe.2010.07.005, PMID: 21056439

[31] Gonzalez-Alvarez A Kimmel KA Rosenkranz SK Mailey E Rosenkranz RR. Are lifestyle behaviors associated with excellent self-rated health among American adolescents? A cross-sectional study. J Healthy Eat Act Living. (2023) 3:112–23. doi: 10.51250/jheal.v3i3.66, PMID: 38344455 PMC10854956

[32] Sargent-Cox K Cherbuin N Morris L Butterworth P Anstey KJ. The effect of health behavior change on self-rated health across the adult life course: a longitudinal cohort study. Prev Med. (2014) 58:75–80. doi: 10.1016/j.ypmed.2013.10.017, PMID: 24201091

[33] Brindal E Hendrie GA Freyne J Noakes M. A mobile phone app designed to support weight loss maintenance and well-being (MotiMate): randomized controlled trial. JMIR Mhealth Uhealth. (2019) 7:e12882. doi: 10.2196/12882, PMID: 31486407 PMC6834303

[34] Kwok SWH Wu CST Tong HT Ho CN Leung KL Leung YCP. Effects of the school-based integrated health promotion program with hydroponic planting on green space use and satisfaction, dietary habits, and mental health in early adolescent students: a feasibility quasi-experiment. Front Public Health. (2021) 9:740102. doi: 10.3389/fpubh.2021.740102, PMID: 34631651 PMC8498580

[35] Carlton J Peasgood T Mukuria C Connell J Brazier J Ludwig K. Generation, selection, and face validation of items for a new generic measure of quality of life: the EQ-HWB. Value Health. (2022) 25:512–24. doi: 10.1016/j.jval.2021.12.007, PMID: 35227597

[36] Fitriana N Hutagalung FD Awang Z Zaid SM. Happiness at work: a cross-cultural validation of happiness at work scale. PLoS One. (2022) 17:e0261617. doi: 10.1371/journal.pone.0261617, PMID: 34986180 PMC8730434

[37] Gupta SK. Intention-to-treat concept: a review. Perspect Clin Res. (2011) 2:109–12. doi: 10.4103/2229-3485.83221, PMID: 21897887 PMC3159210

