# Supplementary material for: The effect of youths as change agents on self-rated health status and happiness among adult neighbors: a cluster randomized controlled trial in Sri Lanka
Source: Front Public Health. 2025 Sep 1;13:1649948. doi: 10.3389/fpubh.2025.1649948 (PMC12433992; doi:10.3389/fpubh.2025.1649948)
Supplement: Supplementary file 1 [file Table_1.docx]

**Supplementary Table 1:** Effect of the intervention on self-rated health and happiness among overweight/obese participants (BMI ≥25 kg/m²) with ≥2 kg weight loss in the intervention group (n=63), compared to the entire control group regardless of their BMI or weight change (n=238).

|  | **Intervention group** | | |  | **Control group** | | |  | **OR (95% CI) between group differences at follow-up** | **P^§^ value** |
| --- | --- | --- | --- | --- | --- | --- | --- | --- | --- | --- |
|  | N (%) at baseline | N (%) at follow-up | Mean ± SD change from the baseline^a^ |  | N (%) at baseline | N (%) at follow-up | Mean ± SD change from the baseline^a^ |  |  |  |
| **Self-rated health** | | |  |  |  |  |  |  |  |  |
| Good | 26 (41.3) | 36 (57.1) | 0.25 ± 0.82 |  | 102 (42.9) | 109 (45.8) | 0.07 ± 0.75 |  | 1.80 (0.97―3.34) | 0.06 |
| **Happiness in the past months** | | | |  |  |  |  |  |  |  |
| Happiness | 27 (42.9) | 31 (49.2) | 0.17 ± 0.83 |  | 109 (45.8) | 95 (39.9) | -0.05 ± 0.83 |  | 1.52 (0.86―2.69) | 0.15 |

Note: OR, odds ratios; CI, confidence interval; SD, standard deviation.

^a^Change from baseline=outcome values at the end of the follow-up ― outcome values at baseline.

^§^ Based on logistic regression analysis, assigning an ordinal number to the categories of self-rated health and happiness.

**Supplementary Table 2:** Effect of intervention on self-rated health and happiness among those who showed positive changes in vegetable or fruit intake from baseline to the endline in the intervention group (n=139), compared to the entire control group (n=238)

|  | **Intervention group** | | |  | **Control group** | | |  | **OR (95% CI) between group differences at follow-up** | **P^§^ value** |
| --- | --- | --- | --- | --- | --- | --- | --- | --- | --- | --- |
|  | N (%) at baseline | N (%) at follow-up | Mean ± SD change from the baseline^a^ |  | N (%) at baseline | N (%) at follow-up | Mean ± SD change from the baseline^a^ |  |  |  |
| **Self-rated health** | | |  |  |  |  |  |  |  |  |
| Good | 52 (37.4) | 81 (58.3) | 0.22 ± 0.92 |  | 102 (42.9) | 109 (45.8) | 0.07 ± 0.75 |  | **2.01 (1.26―3.19)** | **0.003** |
| **Happiness in the past months** | | |  |  |  |  |  |  |  |  |
| Happiness | 54 (38.8) | 72 (51.8) | 0.14 ± 0.90 |  | 109 (45.8) | 95 (39.9) | -0.05 ± 0.82 |  | **1.73 (1.13―2.67)** | **0.01** |

Note: OR, odds ratios; CI, confidence interval; SE, standard deviation.

^a^Change from baseline=outcome values at the end of the follow-up ― outcome values at baseline.

^§^ Based on logistic regression analysis, assigning an ordinal number to the categories of self-rated health and happiness.

**Supplementary Table 3:** Effect of intervention on good self-rated health among those who had poor self-rated health at baseline (intervention group=157; control group=136)

|  | **N (%) at follow-up** | | **OR (95% CI) between-group differences at follow-up** | **P^§^ value** |
| --- | --- | --- | --- | --- |
|  | **Intervention group** | **Control group** |  |  |
| **Self-rated Health** | |  |  |  |
| Good | 70 (44.6) | 35 (25.7) | **2.32 (1.41 ― 3.82)** | **0.001** |

Note: OR, odds ratios; CI, confidence interval.

^§^ Based on logistic regression analysis, assigning an ordinal number to the categories of self-rated health.

**Supplementary Table 4:** Effect of intervention on happiness among those who were unhappy at baseline (intervention group=151; control group=129)

|  | **N (%) at follow-up** | | **OR (95% CI) between-group differences at follow-up** | **P^§^ value** |
| --- | --- | --- | --- | --- |
|  | **Intervention group** | **Control group** |  |  |
| **Happiness in the past months** | |  |  |  |
| Happiness | 56 (37.1) | 39 (30.2) | 1.35 (0.75 ― 2.41) | 0.32 |

Note: OR, odds ratios; CI, confidence interval.

^§^ Based on logistic regression analysis, assigning an ordinal number to the categories of happiness.
